# Supplementary material for: GBP1 recruitment to actin-rich pedestals of extracellular Gram-negative bacteria promotes pyroptosis
Source: EMBO J. 2026 Jun 9;45(14):5046–78. doi: 10.1038/s44318-026-00830-z (PMC13372814; doi:10.1038/s44318-026-00830-z)
Supplement: Supplementary file 2 — Expanded View Figures [file 44318_2026_830_MOESM2_ESM.pdf]

Expanded View Figures

Figure EV1. GBP1 is recruited to actin-rich pedestals of attaching and effacing (A/E) pathogens.

(**A**) Graph showing percentage pyroptotic cell death (top) and representative immunoblots (below) of HeLa cells stably expressing non-targeting (negative control; CTRL) or miR30E targeting caspase-4 (*CASP4*) or gasdermin D (*GSDMD*). Graph (top) shows percentage propidium iodide dye uptake assays in the indicated cells infected with EPEC for 6 h. Cells were primed with IFNγ (10 ng.mL^{-1}) 16 h before infection or preparation of cell lysates for western blots. Mean ± SD error bars with symbols representing data from $n = 3$ independent experiments are shown. **$P < 0.01$, two-tailed P values for comparisons of CTRL and the indicated miRNA-expressing cells from mixed effects ANOVAs ($P = 0.0037$ for the two indicated comparisons). Representative immunoblots (below) of caspase-4, GSDMD and GAPDH (loading control) from $n = 3$ independent experiments. (**B**) ELISA quantification of IL-18 from supernatants of IFNγ-primed HeLa cells of the indicated genotypes infected with wild-type (WT) or Δ*escF* mutant of EPEC for 6 h. Mean ± SD error bars with symbols representing data from $n = 3$ independent experiments are shown. ***$P < 0.001$ ($P = 2.6$e-07) is a two-tailed P value for the indicated comparisons from mixed effects ANOVAs. *nd*, not detected. (**C**) Ribbon structures of "extended" or "open" crossover dimer of GBP1 on the left (adapted from PDB: 8R1A), and "closed" or "safety pin" dimer (modelled on PDB: 2B92 LG dimer bound to GDP.AlF_3) shown on the right. GBP1 monomers coloured pastel shades of cyan and wheat, respectively. Residues K51, C589, M139, ^{308}DLP^{310} linker, GDP.AlF_3 and the farnesyl group are highlighted. C-terminal sequences for GBP1 and GBP2 are described with CaaX-box motifs, and the GBP1 polybasic RRR motif is shown in bold. The prenylation state of the protein is indicated with F or GG (farnesyl and geranylgeranyl, respectively). (**D**) Representative immunoblots showing the expression of the indicated tetracycline (Tet)-controlled WT GBP1 or the indicated variants in *GBP1*$^{-/-}$ cells. Cells were stably transduced with expression plasmids for the indicated proteins, treated with IFNγ (10 ng.mL^{-1}) and doxycycline (200 ng.mL^{-1}) for 16 h and cell lysates were prepared for western blots. Data are representative of $n = 2$ independent experiments. Images shown are cropped from the same membrane at the same exposure to remove unnecessary lanes. (**E**) ELISA quantification of IL-18 from supernatants of IFNγ- and doxycycline-treated wild-type (WT) or *GBP1*$^{-/-}$ HeLa cells stably expressing mCherry2 (C), mCherry2-tagged WT GBP1 or K51A or C589A mutants, or mVenus (V)-GBP2 or variants as indicated. Mean ± SD error bars with symbols representing data from $n = 3$ independent experiments are shown. ns, not significant; ***$P < 0.001$, two-tailed P values for comparisons between WT cells and cells expressing the indicated GBP1 or GBP2 variants from mixed effects ANOVAs. Exact P values as follows—C: 1.7e-06; cGBP1: 0.9282; ^{G}GBP1^{K51A}: 5.5e-10; ^{G}GBP1^{C589A}: 5.5e-10; ^{V}GBP2: 1.8e-09; ^{V}GBP2^{CTIS}: 7.8e-10; ^{V}GBP2^{3RCTIS}: 3.2e-08.

© The Author(s)

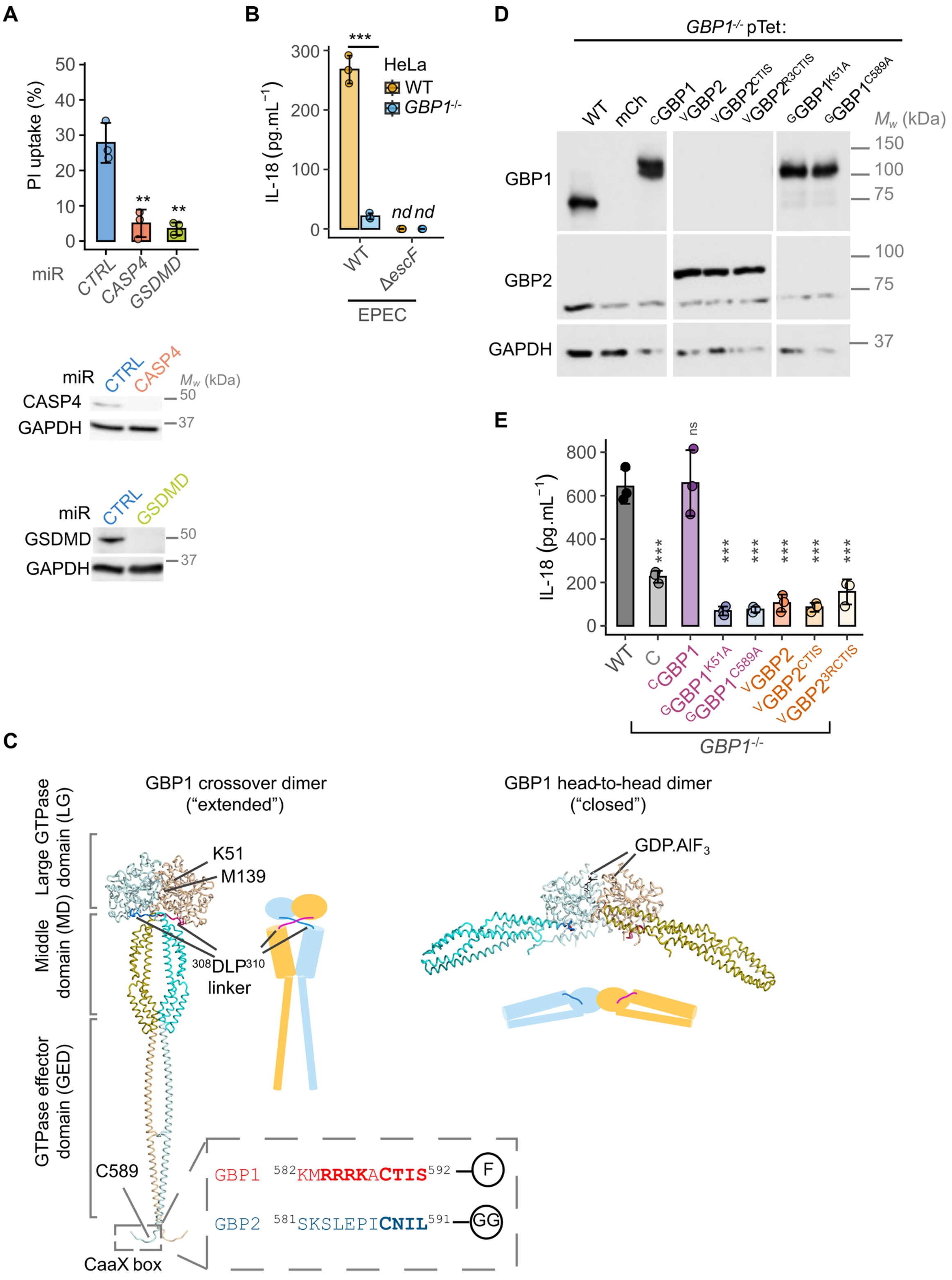
A
PI uptake (%)
miR
CTRL
CASP4
GSDMD
CASP4
GAPDH
GSDMD
M_w (kDa)
50
37
B
IL-18 (pg.mL^{-1})
HeLa
WT
GBP1-/-
nd nd
WT
ΔescF
EPEC
D
GBP1-/- pTet:
WT
mCh
CGBP1
VGBP2
VGBP2CTIS
VGBP2R3CTIS
GGBP1K51A
GGBP1C589A
M_w (kDa)
GBP1
GBP2
GAPDH
150
100
75
37
E
IL-18 (pg.mL^{-1})
ns
GBP1-/-
C
GBP1 crossover dimer
("extended")
GBP1 head-to-head dimer
("closed")
Large GTPase domain (LG)
Middle domain (MD)
GTPase effector domain (GED)
K51
M139
308DLP310 linker
GDP.AlF3
C589
CaaX box
GBP1 582KMRRRKACTIS592 F
GBP2 581SKSLEPICNIL591 GG

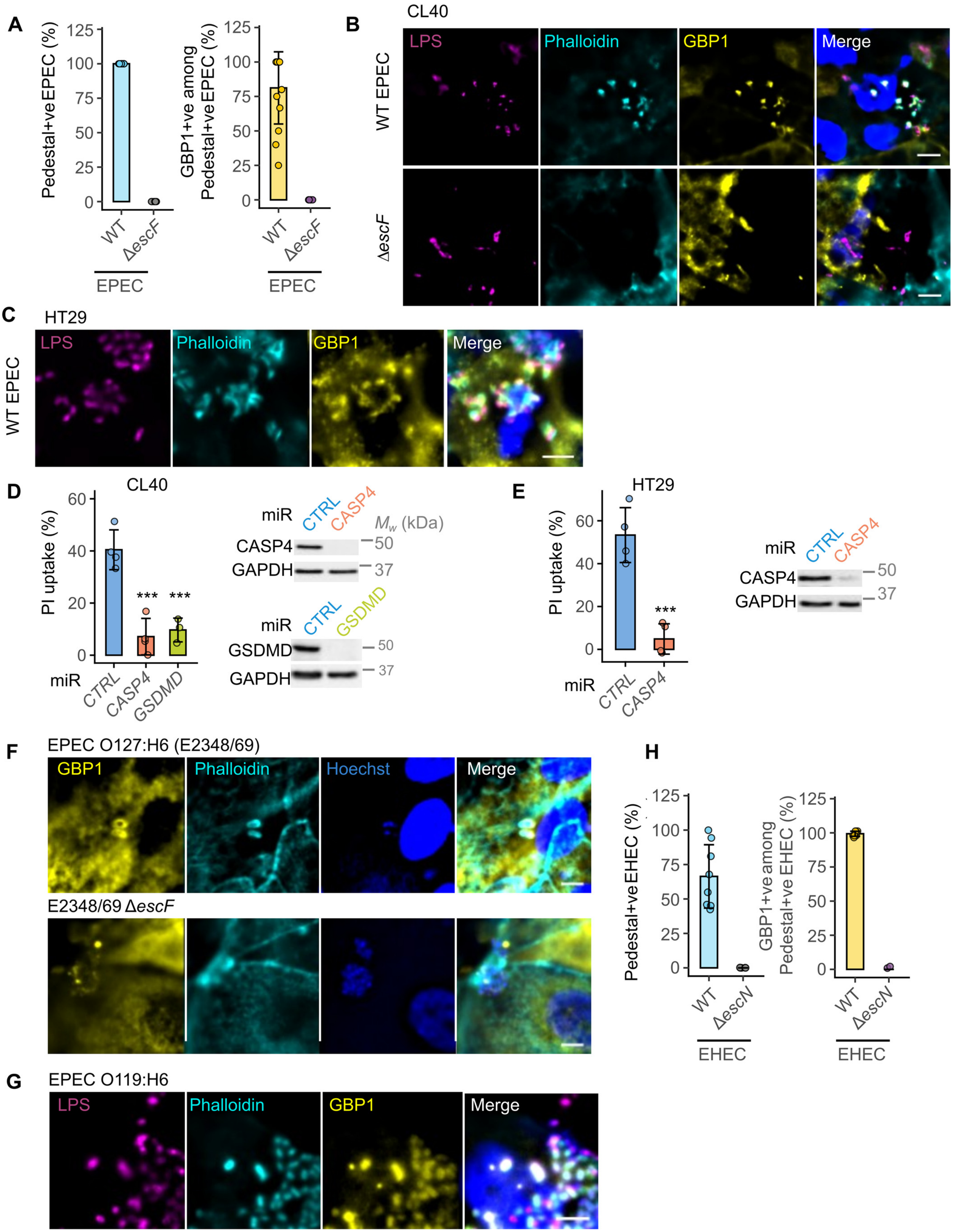
A
Pedestal+ve EPEC (%)
125
100
75
50
25
0
WT
ΔescF
EPEC
GBP1+ve among Pedestal+ve EPEC (%)
B
CL40
LPS
Phalloidin
GBP1
Merge
WT EPEC
ΔescF
C
HT29
D
CL40
PI uptake (%)
60
40
20
***
miR
CTRL
CASP4
GSDMD
M_w (kDa)
GAPDH
50
37
E
HT29
H
Pedestal+ve EHEC (%)
GBP1+ve among Pedestal+ve EHEC (%)
ΔescN
EHEC
F
EPEC O127:H6 (E2348/69)
Hoechst
E2348/69 ΔescF
G
EPEC O119:H6

Figure EV2. GBP1 is recruited to actin-rich attachment sites of extracellular EPEC.

(**A**) Quantification of microcolonies of wild-type (WT) or Δ*escF* mutant of EPEC infected in IFNγ-primed HeLa cells staining positive for actin-rich pedestals observed with phalloidin staining (Pedestal+ve) or positive for both phalloidin and GBP1 (GBP1+ve among Pedestal+ve) at 2 h post-infection (also see images in Fig. 1). Mean ± SD error bars with symbols representing individual fields of view from $n = 3$ independent experiments are shown. (**B**) Representative immunofluorescence images of IFNγ-primed human CL40 colonic epithelial cells infected with WT EPEC or Δ*escF* mutant for 1 h. Cells were stained with anti-LPS and anti-GBP1 antibodies, phalloidin-Alex568 (to stain actin) and Hoechst (DNA dye; blue). Data are representative of $n = 3$ independent experiments. Scale bar, 5 µm. (**C**) Representative immunofluorescence images of IFNγ-primed HT29 cells infected with EPEC E2348/69 for 3 h. Cells were stained with anti-GBP1 antibodies, phalloidin-Alex568 (to stain actin) and Hoechst (DNA dye; blue). Data from $n = 2$ independent experiments. Scale bar, 5 µm. (**D**) Graph showing percentage pyroptotic cell death (left) at 6 h post-infection with EPEC and representative immunoblots (right) of CL40 cells stably expressing negative control (CTRL) or miR30E targeting caspase-4 (*CASP4*) or gasdermin D (*GSDMD*). Cells were primed with IFNγ (10 ng.mL^{-1}) 16 h before infection. Mean ± SD error bars with symbols representing data from $n = 3$ independent experiments are shown. ***$P < 0.001$, two-tailed *P* values for comparisons between CTRL and indicated miRNA-expressing cells from mixed effects ANOVAs ($P = 0.0008$ for the two indicated comparisons). Representative immunoblots (right) of caspase-4, GSDMD and GAPDH (loading control) from $n = 3$ independent experiments. (**E**) Graph showing percentage pyroptotic cell death (left) at 12 h post-infection with EPEC and representative immunoblots (right) of HT29 cells stably expressing negative control (CTRL) or miR30E targeting caspase-4 (*CASP4*). Cells were primed with IFNγ (10 ng.mL^{-1}) 16 h before infection. Mean ± SD error bars with symbols representing data from $n = 4$ independent experiments are shown. ***$P < 0.001$, two-tailed *P* values for comparisons between CTRL and *CASP4*-miRNA-expressing cells from mixed effects ANOVAs are indicated ($P = 9.1e\text{-}07$). Representative immunoblots (right) of caspase-4 and GAPDH (loading control) from $n = 3$ independent experiments. (**F**) Representative immunofluorescence images of IFNγ-primed 2D monolayers prepared from human colonic organoids infected with wild-type EPEC or Δ*escF* mutant for 2 h as indicated. Cells were stained with anti-GBP1 antibodies, phalloidin (to stain actin) and Hoechst (DNA dye; blue) as labelled. Scale bar, 5 µm. (**G**) Representative immunofluorescence images of IFNγ-primed HeLa infected with the indicated strain of EPEC for 3 h. Cells were stained with anti-LPS and anti-GBP1 antibodies, phalloidin (to stain actin) and Hoechst (DNA dye; blue). Data are representative of $n = 3$ independent experiments. Scale bar, 5 µm. (**H**) Quantification of wild-type (WT) or Δ*escN* mutant of EHEC infected in IFNγ-primed HeLa cells, staining positive for actin-rich pedestals observed with phalloidin staining (Pedestal+ve) or positive for both phalloidin and GBP1 (GBP1+ve among Pedestal+ve) at 5 h post-infection (also see images in Fig. 1). Mean ± SD error bars with symbols representing individual fields of view from $n = 3$ independent experiments are shown.

 © The Author(s)

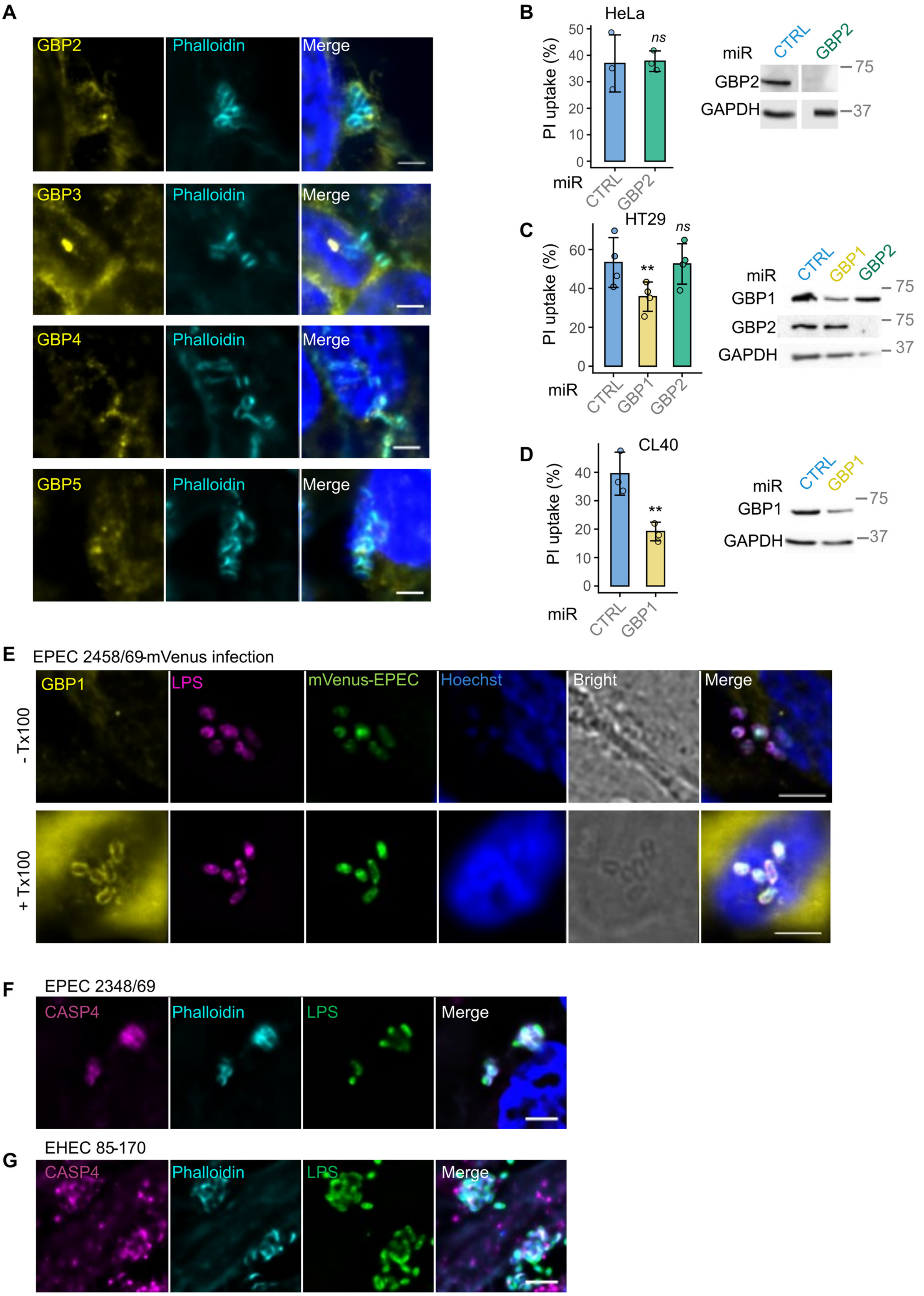
A
GBP2
Phalloidin
Merge
GBP3
Phalloidin
Merge
GBP4
Phalloidin
Merge
GBP5
Phalloidin
Merge
B
HeLa
PI uptake (%)
ns
miR
CTRL
GBP2
GBP2
GAPDH
75
37
C
HT29
PI uptake (%)
**
ns
miR
CTRL
GBP1
GBP2
GBP1
GBP2
GAPDH
75
75
37
D
CL40
PI uptake (%)
**
miR
CTRL
GBP1
GBP1
GAPDH
75
37
E
EPEC 2458/69-mVenus infection
GBP1
LPS
mVenus-EPEC
Hoechst
Bright
Merge
- Tx100
+ Tx100
F
EPEC 2348/69
CASP4
Phalloidin
LPS
Merge
G
EHEC 85-170
CASP4
Phalloidin
LPS
Merge

Figure EV3. GBP1 is recruited to actin-rich attachment sites of extracellular EPEC.

(**A**) Representative immunofluorescence images of IFNγ-primed and doxycycline-treated HeLa cells showing the expression of the indicated GBPs and infected with EPEC for 3 h. Cells were fixed, and ^{FLAG}GBP2, ^{FLAG}GBP3, ^{MYC}GBP4 and ^{FLAG}GBP5 were stained with anti-FLAG or anti-MYC antibodies, phalloidin (to stain actin) and Hoechst (DNA dye; blue). Data are representative of $n = 3$ independent experiments. Scale bar 4 µm. (**B**) Graph showing percentage pyroptotic cell death (left) at 6 h post-infection with EPEC and representative immunoblots (right) of HeLa cells stably expressing negative control (CTRL) or miR30E targeting *GBP2*. Cells were primed with IFNγ (10 ng.mL^{-1}) 16 h before infection. Mean ± SD error bars with symbols representing data from $n = 3$ independent experiments are shown. *ns*, not significant ($P = 0.8541$) for comparison between CTRL and *GBP2* miRNA-expressing cells from mixed effects ANOVAs. Representative immunoblots (right) of GBP2 and GAPDH (loading control) from $n = 3$ independent experiments. Images show the same exposures of the same blots for GBP2 and GAPDH with irrelevant lanes removed. (**C**) Graph showing percentage pyroptotic cell death (left) at 12 h post-infection with EPEC and representative immunoblots (right) of HT29 cells stably expressing negative control (CTRL) or miR30E targeting *GBP1* or *GBP2*. Cells were primed with IFNγ (10 ng.mL^{-1}) 16 h before infection. Mean ± SD error bars with symbols representing data from $n = 4$ independent experiments are shown. $^{**}P = 0.0014$; *ns*, not significant ($P = 0.8417$) are two-tailed *P* values for comparison of CTRL with the indicated miRNA-expressing cells from mixed effects ANOVAs. Representative immunoblots (right) of GBP1, GBP2 and GAPDH (loading control) from $n = 4$ independent experiments. (**D**) Graph showing percentage pyroptotic cell death (left) at 6 h post-infection with EPEC and representative immunoblots (right) of CL40 cells stably expressing negative control (CTRL) or miR30E targeting *GBP1*. Cells were primed with IFNγ (10 ng.mL^{-1}) 16 h before infection. Mean ± SD error bars with symbols representing data from $n = 3$ independent experiments are shown. $^{**}P = 0.0079$ is a two-tailed *P* values for comparisons between CTRL and *GBP1* miRNA-expressing cells from mixed effects ANOVAs. Representative immunoblots (right) of GBP1 and GAPDH (loading control) from $n = 3$ independent experiments. (**E**) Representative immunofluorescence images of IFNγ-primed HeLa cells infected with EPEC-mVenus for 1 h and then fixed and stained without and with permeabilisation as labelled. Cells were fixed and either permeabilised for 3 min with 0.3% Triton X100 or not prior to staining with anti-LPS and anti-GBP1 antibodies, and Hoechst (DNA dye; blue). Data are representative of $n = 3$ independent experiments. Scale bar, 5 µm. (**F**, **G**) Representative immunofluorescence images of IFNγ-primed HeLa cells infected with EPEC 2348/69 for 2 h (**A**) or EHEC 85–170 for 5 h (**B**) showing endogenous caspase-4 trafficking to actin-rich pedestals induced by bacteria. Cells were stained with anti-LPS (to stain bacteria) and anti-caspase-4 antibodies, phalloidin (to stain actin), and Hoechst (DNA dye; blue). Data are representative of $n = 2$ independent experiments. Scale bar, 5 µm.

 © The Author(s)

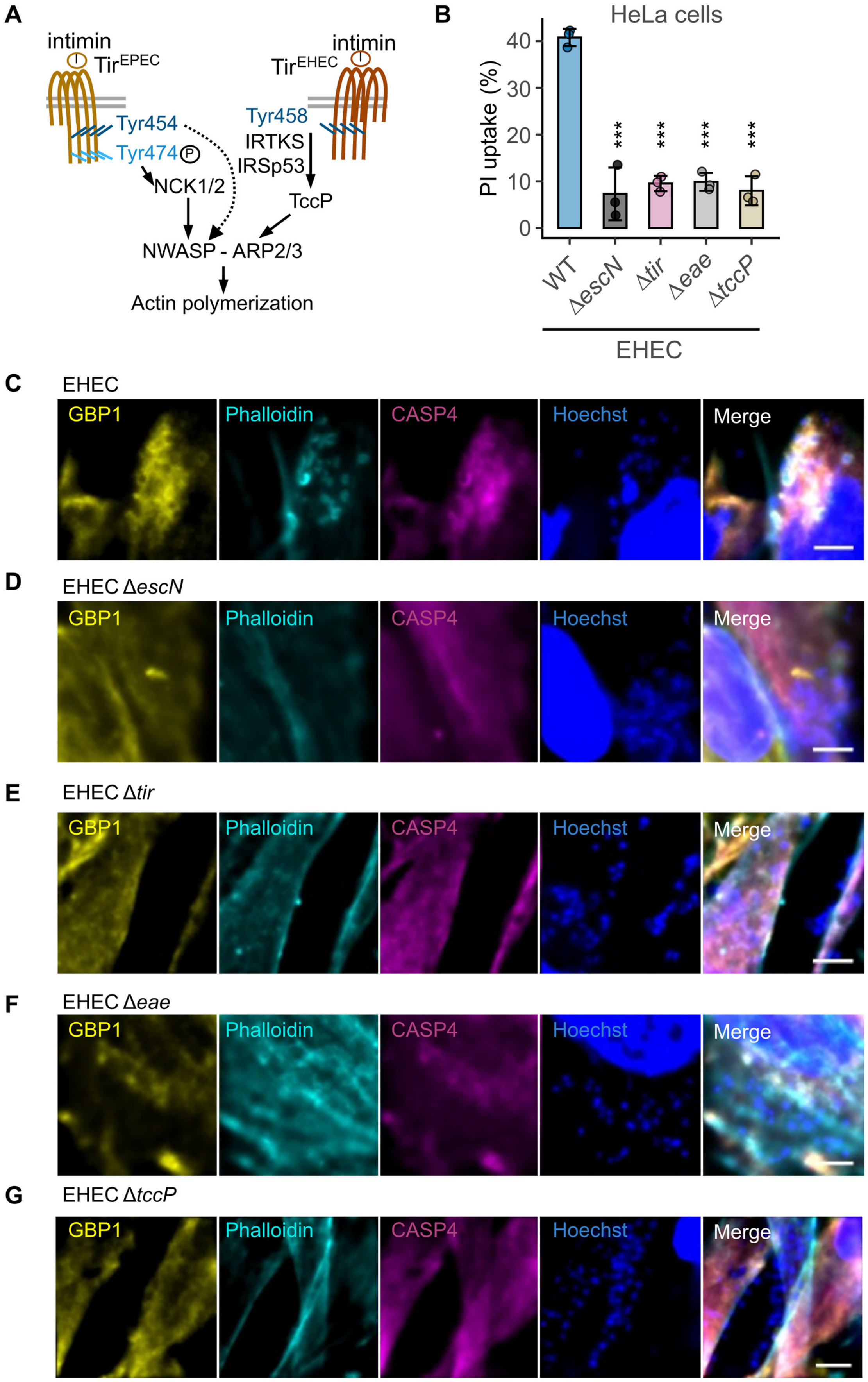
A
intimin
Tir^EPEC
Tyr454
Tyr474 P
NCK1/2
intimin
Tir^EHEC
Tyr458
IRTKS
IRSp53
TccP
NWASP - ARP2/3
Actin polymerization
B
HeLa cells
PI uptake (%)
0
10
20
30
40
***
WT
ΔescN
Δtir
Δeae
ΔtccP
EHEC
C
EHEC
GBP1
Phalloidin
CASP4
Hoechst
Merge
D
EHEC ΔescN
E
EHEC Δtir
F
EHEC Δeae
G
EHEC ΔtccP

Figure EV4. Tir-intimin signalling is essential for GBP1-caspase-4 recruitment to actin-rich pedestals of EHEC.

(**A**) A schematic showing the distinct pathways of actin polymerisation driven by Tir^{EPEC} and Tir^{EHEC}. Tir^{EPEC} mainly relies on Y474 tyrosine phosphorylation, NCK recruitment and N-WASP-ARP2/3-dependent actin polymerisation. Tir^{EHEC} uses Y458 to recruit IRTKS/IRSp53, which bridge the interaction between Tir, TccP and N-WASP-ARP2/3. The Y454 residue in Tir^{EPEC} can also recruit IRTKS/IRSp53, but this does not lead to actin-rich structures due to the lack of TccP in EPEC 2348/69 (shown by dotted arrow). (**B**) Percentage pyroptotic cell death as measured by propidium iodide dye uptake assays of IFNγ-primed HeLa cells infected with wild-type EHEC (WT) or the indicated mutants for 8 h. Mean ± SD error bars with symbols representing data from $n = 3$ independent experiments are shown. ***$P < 0.001$, two-tailed P values for comparisons of the indicated mutant strains with the WT from mixed effects ANOVAs. P values as follows – Δ*escN*: 1.6e-08; Δ*tir*: 1.9e-08; Δ*eae*: 1.9e-08; Δ*tccP*: 1.6e-08. (**C–G**) Representative immunofluorescence images of IFNγ-primed HeLa cells expressing YFP-$Caspase4^{C285S}$ infected with wild-type EHEC or the mutants for 5 h. Cells were stained with anti-GBP1 antibodies, phalloidin-Alex568 (to stain actin) and Hoechst (DNA dye; blue). Data from $n = 3$ independent experiments. Scale bar, 5 µm.

 © The Author(s)

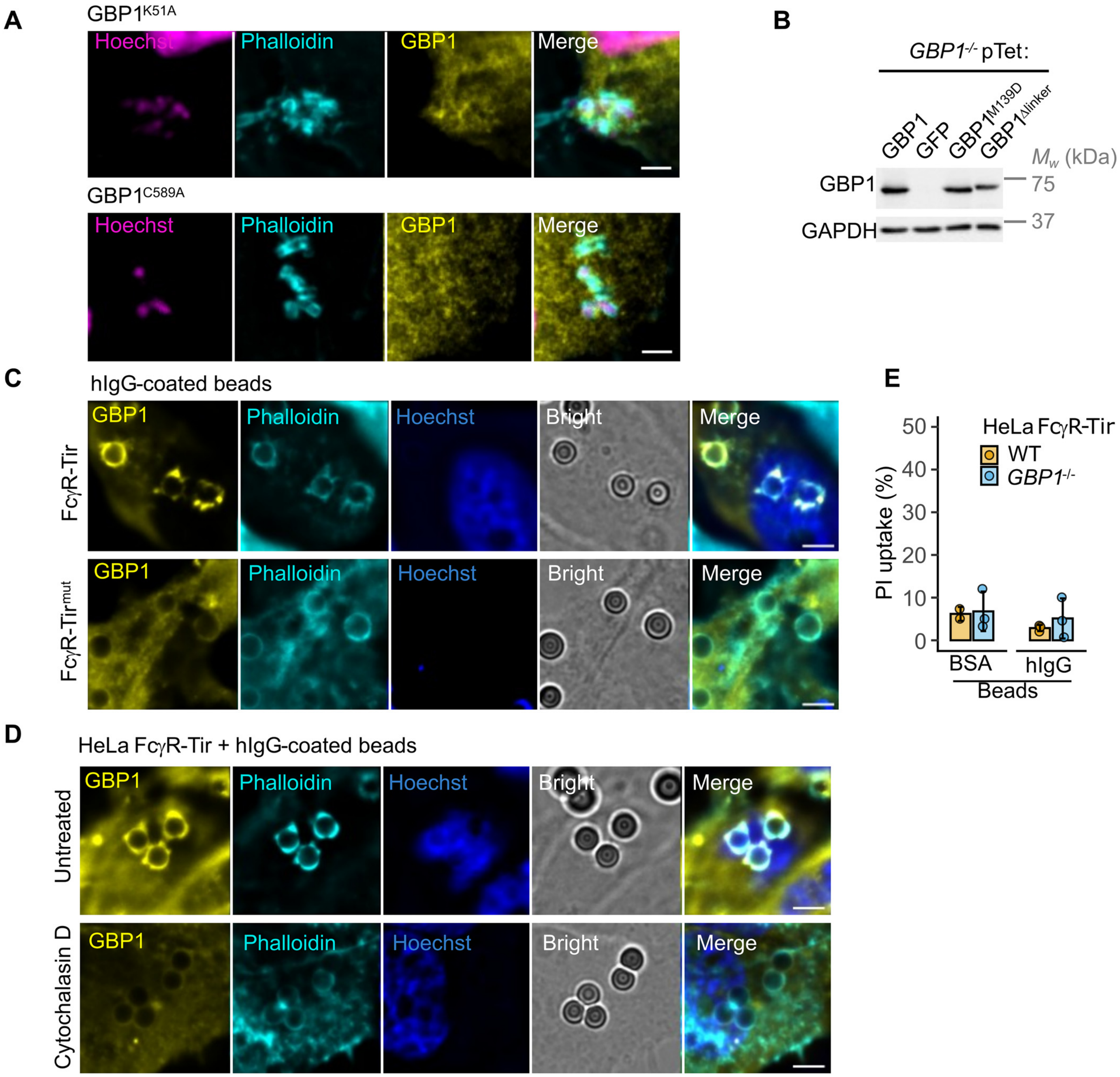

Figure EV5. Inhibition of actin polymerisation by FcγR-Tir fusion protein blocks GBP1 recruitment to beads.

(**A**) Representative images from IFNγ-primed *GBP1*$^{-/-}$ expressing GFP-tagged GBP1 K51A or C589A as indicated and infected with EPEC for 3 h. Cells were stained with anti-GBP1, phalloidin (to stain actin) and Hoechst (DNA dye for nuclei and EPEC; magenta). Scale bar, 4 μm. Data from $n = 3$ independent experiments. (**B**) Representative immunoblots of the indicated tetracycline-controlled WT or GBP1 variants in *GBP1*$^{-/-}$ cells. Cells were stably transduced with expression plasmids for the indicated proteins, treated with IFNγ (10 ng.mL^{-1}) and doxycycline (200 ng.mL^{-1}) for 16 h and cell lysates were prepared for western blots. Data are representative of $n = 2$ independent experiments. (**C**) Representative images from HeLa cells expressing FcγR-Tir or FcγR-Tir^{mut} (with C-terminal mCherry2 tag) treated for 3 h with sterile polystyrene beads coated with hIg. Cells were stained with an anti-GBP1 antibody, phalloidin (to stain actin) and Hoechst (DNA dye; blue). Data are representative of $n = 3$ independent experiments. Scale bar, 5 μm. (**D**) Representative images from HeLa cells expressing FcγR-Tir (with C-terminal mCherry2 tag) treated for 3 h with sterile polystyrene beads coated with hIg in the absence or presence of the actin polymerisation inhibitor cytochalasin D (100 nM). Cells were stained with an anti-GBP1 antibody, phalloidin (to stain actin) and Hoechst (DNA dye; blue). Data are representative of $n = 3$ independent experiments. Scale bar, 5 μm. (**E**) Percentage pyroptotic cell death as measured by propidium iodide dye uptake assays of IFNγ-primed HeLa cells of the indicated genotypes stably expressing FcγR-Tir cells treated for 6 h with BSA or hIgG-coated beads as indicated. Mean ± SD error bars with symbols representing data from $n = 5$ independent experiments are shown.

© The Author(s)
