# Supplementary material for: GBP1 recruitment to actin-rich pedestals of extracellular Gram-negative bacteria promotes pyroptosis
Source: EMBO J. 2026 Jun 9;45(14):5046–78. doi: 10.1038/s44318-026-00830-z (PMC13372814; doi:10.1038/s44318-026-00830-z)
Supplement: Supplementary file 1 — Peer Review File [file 44318_2026_830_MOESM1_ESM.pdf]

THE EMBO JOURNAL

GBP1 recruitment to actin-rich pedestals of extracellular Gram-negative bacteria promotes pyroptosis

Daniel Bennison, Ishaan Chaudhary, Dharitri Chaudhuri, Justin Chun Ngai Wong, Ayush Punwatkar, Priyanka Biswas, Miyu Stephenson, Qiyun Zhong, Wouter Kallemeijn, Marianne Guenot, Sandra Koigi, Diana Papp, John Thomas, Dimple Dixit, Tamas Korcsmaros, Arthur Talman, Eva-Maria Frickel, Edward Tate, Sandhya Visweswariah, Gad Frankel, and Avinash Shenoy

Corresponding author(s): Avinash Shenoy (a.shenoy@imperial.ac.uk)

Review Timeline:

Submission Date:	25th Sep 25
Editorial Decision:	11th Nov 25
Appeal Received:	25th Mar 26
Editorial Decision:	28th Apr 26
Revision Received:	8th May 26
Accepted:	15th May 26

Editor: Ioannis Papaioannou

Transaction Report:

(Note: With the exception of the correction of typographical or spelling errors that could be a source of ambiguity, letters and reports are not edited. Depending on transfer agreements, referee reports obtained elsewhere may or may not be included in this compilation. Referee reports are anonymous unless the Referee chooses to sign their reports.)

Dear Avinash,

Thank you again for submitting your manuscript EMBOJ-2025-122562 to The EMBO Journal for our consideration, and for your patience during peer review. Your manuscript has now been seen by three experts in the field, and we have received the full set of their comments, which you can find appended below.

As you will see, the reviewers indicate interest in your study, and reviewer #3 in particular is very supportive of the manuscript. However, referees #1 and #2 identify a number of major limitations and weaknesses, which together bring the novelty, depth/advance when it comes to the mechanistic understanding of the new observations, and physiological relevance of the findings into question.

I regret to have to say that, in light of these comments and concerns raised by the referees, our conclusion was that the manuscript is not sufficiently developed for further consideration at The EMBO Journal.

Having said that, I would like to point out that we are very interested in this area of research and the topic of the manuscript in particular, and we would be open to reconsidering a thoroughly revised version of the manuscript should you be willing to embark on a major revision and obtain in the near future more data that would adequately address the limitations and concerns raised by the referees. I should add, however, that it would be treated as a new submission, which means that all new literature at the time of resubmission would have to be taken into account in our consideration of the provided conceptual advance. Please let me know if you have any questions you might want to discuss with me.

I am sorry we have to disappoint you on this occasion, but I hope that the comments of the referees will help you strengthen your work further, and that we might have the opportunity to reconsider a substantially strengthened version in the near future. I would like to thank you once again for your interest in our journal and the opportunity to consider your findings.

Best regards,

Ioannis

Ioannis Papaioannou, PhD
Editor, The EMBO Journal
i.papaioannou@embojournal.org

****

Referee #1:

In this study, Bennison and colleagues describe an intriguing mechanism of GBP1 and Caspase-4 recruitment to actin-enriched pedestals induced by TIR-encoded bacteria. Subsequently, recruited caspase-4 is activatd by LPS which then trigger cell pyroptosis and IL-18 release in epithelial cells.
The findings are of interest and the ability of actin to recruit both GBP1 and Caspase-4 is novel.

MAJOR points:
However some parts would require additional experiments or tempering some conclusion to validate the authors interpretations regarding:
1/ GBP-1-driven Caspase-4 recruitment. While there is a decrease of Casp4 recruitment in actin pedestal in GBP1 KO cells, a lot of Casp4 is still recuited to these actin structures in absence of GBP1. Is there an involvement of other GBPs? a GBP-independent way of recruiting Casp4 in actin pedestals? Experiments with various GBP KOs or discussion on dampening a bit the central involvement of GBP1 as the central inducer of Casp4 in these strctures should be done as the author' conclusions do not fit with the results presented.
2/ If GBP-1-dependent and indepndent recruitment of Casp4 to actin pedestal requires actin polymerization (either induced by bacteria or by the Ig-TIR constructs), one should expect that GBP1 and Casp4 would also target actin-comet tails from gram-positive bacteria Listeria monocytogenes or would also be able to target nacent phagosomes on macrophages as both are extremely enriched in polymerized actin structures that ressemble the pedestals (podosome-like structures or actin tails). These results would streghthen the author findings on GBP1 and Casp4 recruitment on these actin structures.
3/ The results that actin can recruit both GBP1 and Casp4 is of strong interest. Yet, at this step, there is a lack of discussion/ result about the putative function of GBP1 and Casp4 on actin structures that would be independent from the LPS-driven non

canonical inflammasome response. I understand that authors might not have the experimental answer yet, but having some broader dicussion/speculation on this would be a strong added value for the study.

Referee #2:

This manuscript by Shenoy and colleagues examines how extracellular A/E pathogens (EPEC, EHEC, Citrobacter rodentium) triggers pyroptosis in epithelial cells in humans and mice. Here, the authors demonstrate that bacterial-induced actin polymerisation triggers sequential recruitment of GBP1 and caspase-4 and downstream epithelial cell pyroptosis. While the observation that actin polymerisation is sufficient to induce GBP1 and caspase-4 recruitment in the absence of LPS is surprising, the overall conceptual advancement of the remaining manuscript is incremental (points 1, 2, 4) and contradictory to the authors' earlier work (point 3). I have also listed other suggestions for the authors' consideration (point 5 onwards).

1. The authors have already demonstrated in their 2019 Cell Reports paper (PMID: 31018119) that EPEC-induced actin polymerisation triggers caspase-4 activation in human macrophages, thus, demonstrating that the same pathway occurs in epithelial cells is not conceptually novel.
2. The requirement for GBP1 to activate caspase-4 is well established and was demonstrated by the authors themselves and several other groups. Non-exhaustive list - PMID: 32783936, 31268602, 32581219, 32541830, 32510692, 37023136.
3. A previous study from these authors demonstrated that Tir clustering without downstream actin polymerisation is sufficient to trigger epithelial cell pyroptosis (PMID: 33378358), yet in this current manuscript the same group of authors report that actin polymerisation is required for GBP1 and caspase-4 recruitment and downstream pyroptosis. These results are contradictory to one another.
4. The most interesting (and novel) observation from this study is that GBP1 senses A/E-pathogen-induced actin polymerisation. However, this finding is mostly descriptive and the mechanism that drives GBP1 recruitment remains unclear.
5. Majority of the work was done using HeLa cell, a transformed cervical epithelial cell line. Since A/E pathogens infect IECs, this work should be performed in IECs to reflect physiological relevance.
6. I am assuming Hela (and IEC cell lines) do not express NLRP3 and thus IL-18 cleavage is caspase-4-dependent but not through secondary NLRP3-caspase-1 activation? This should be experimentally validated.
7. The authors demonstrated GBP1 and caspase-4-dependency in driving pyroptosis but has assumed in the manuscript that the downstream effector is GSDMD. Is this the case? This should be validated experimentally with GSDMD knock out and GSDMD cleavage.
8. The use of anti-LPS to detect Citrobacter rodentium in murine colon is far from ideal, since anti-LPS will pick up all Gram-negative bacteria from the colon. Using a more specific antibody will be more appropriate and convincing.

Referee #3:

The interferon-induced GTPase guanylate-binding protein 1 (GBP1) is well established as a key mediator of noncanonical inflammasome activation, specifically involving caspase-4 (CASP4) in humans. GBP1 has been shown to trigger CASP4 activation in response to infection with intracellular Gram-negative bacterial pathogens. Mechanistically, three principal models have been proposed, all converging on GBP1's capacity to interact with lipopolysaccharide (LPS):
i) GBP1 encapsulates Gram-negative bacteria in a detergent-like manner, promoting the release of LPS into the cytosol where it becomes available for CASP4 recognition
ii) GBP1 disrupts the O-antigen barrier, thereby exposing the lipid A moiety of the bacterial outer membrane to direct CASP4 binding
iii) GBP1 facilitates the aggregation of cytosolic LPS, forming high-affinity LPS platforms that enhance CASP4 recruitment.
However, these models have not adequately accounted for the phenomenon of CASP4 activation induced by extracellular Gram-negative bacteria. The present study addresses this gap and introduces a significant conceptual advance by revealing an LPS-independent mechanism of CASP4 recruitment mediated by actin pedestal formation.
Specifically, this study demonstrates that GBP1 is targeted to actin-pedestal structures formed by enteropathogenic (EPEC) and enterohaemorrhagic (EHEC) E. coli, and the EPEC-like murine pathogen Citrobacter rodentium, both in human cells and in vivo (mouse colonocytes). It is shown that GBP1 localizes to actin pedestals generated by Tir-Intimin signaling, a key virulence mechanism of EPEC/EHEC. Importantly, it is shown that this recruitment occurs independent of bacterial LPS. Using a chimeric receptor (FcR-Tir) that mimics Tir-driven actin polymerization without bacteria, the study proves that actin remodeling alone is sufficient to drive GBP1 recruitment, separating this process from LPS-dependent signaling. The findings extend GBP1's role from pattern recognition of cytosolic LPS to a novel effector-triggered immunity (ETI) function, where GBP1 acts as a sentinel for pathogen-induced actin cytoskeleton changes. The design of the studies is rigorous throughout. The paper is well written and flows logically. I don't see any notable weaknesses and am convinced that the paper will have a substantial impact on field. While the study is already quite comprehensive, there appears to be a minor yet notable gap in the proposed model. The paper convincingly demonstrates that CASP4 is recruited to actin pedestals in an LPS-independent but GBP1-dependent manner. A direct GBP1-CASP4 protein-protein interaction would provide the most straightforward mechanistic explanation for this observation. Although such experiments are not essential-given that the main conclusions are well supported by the presented

data-it seems likely that the authors have considered, or perhaps even attempted, to explore this possibility. Some elaboration on this point, ideally supported by experimental evidence, would further strengthen the manuscript. In the same vein, it would be valuable for the authors to discuss their findings in the context of previous studies demonstrating GBP1-CASP4 co-recruitment to cytosolic Gram-negative bacteria. The prevailing view posits that GBP1 coating exposes the lipid A portion of LPS, thereby enabling CASP4 binding. However, in light of the current findings, an alternative model - where GBP1 directly recruits CASP4 to bacterial surfaces independently of LPS - merits consideration. Lastly, it would be interesting to discuss whether GBP1 recognition of pathogen-containing vacuoles (PVs) might similarly lead to CASP4 recruitment, or whether GBP1 interactions at actin pedestals represent a fundamentally distinct mode of CASP4 engagement.

** As a service to authors, EMBO Press provides authors with the possibility to transfer a manuscript that one journal cannot offer to publish to another EMBO publication or the open access journal Life Science Alliance launched in partnership between EMBO Press, Rockefeller University Press and Cold Spring Harbor Laboratory Press. The full manuscript and if applicable, reviewers' reports, are automatically sent to the receiving journal to allow for fast handling and a prompt decision on your manuscript. For more details of this service, and to transfer your manuscript please click on Link Not Available. **

Please do not share this URL as it will give anyone who clicks it access to your account.

We thank the reviewers for their constructive comments and have addressed them below with new text (which appears in red colour) as requested or experimental data (new Figure 4 and 12 total new panels of data overall).

Referee #1: MAJOR points:
In this study, Bennison and colleagues describe an intriguing mechanism of GBP1 and Caspase-4 recruitment to actin-enriched pedestals induced by TIR-encoded bacteria. Subsequently, recruited caspase-4 is activatd by LPS which then trigger cell pyroptosis and IL-18 release in epithelial cells. The findings are of interest and the ability of actin to recruit both GBP1 and Caspase-4 is novel.

However some parts would require additional experiments or tempering some conclusion to validate the authors interpretations regarding:
1/ GBP-1-driven Caspase-4 recruitment. While there is a decrease of Casp4 recruitment in actin pedestal in GBP1 KO cells, a lot of Casp4 is still recuited to these actin structures in absence of GBP1.

The reviewer is correct in that occasionally caspase-4 is observed around pedestals. Indeed, we have stated that "Caspase-4 recruitment is reduced in *GBP1*$^{-/-}$ cells..." (line ~223). However, as caspase-4 activation requires GBP1, caspase-4 localised to actin-rich structures is unlikely to be activated in *GBP1*$^{-/-}$ cells. To address this comment we have now added in the discussion (line ~330): "Further, murine caspase-4/11 is proposed to localise to the leading edge in leukocytes; therefore, the diffuse caspase-4 staining at EPEC/EHEC pedestals in *GBP1*$^{-/-}$ cells may point to its ability to traffic to the plasma membrane in homeostasis [57]."

Is there an involvement of other GBPs? a GBP-independent way of recruiting Casp4 in actin pedestals? Experiments with various GBP KOs or discussion on dampening a bit the central involvement of GBP1 as the central inducer of Casp4 in these strctures should be done as the author' conclusions do not fit with the results presented.

We thank the reviewer for this comment. We have addressed it by generating cells expressing tetracycline-inducible Flag-GBP2, Flag-GBP3, Myc-GBP4 and Flag-GBP5, which are the most widely expressed GBPs and previously implicated in caspase-4 activation in some studies. In new data (**in new Figure S3A**) we show that GBP3-5 do not localise to pedestals and GBP2 was found in the vicinity of a minority of pedestals (~20%, whereas GBP1 localises to >75% EPEC pedestals). Therefore, we assessed the role of GBP2 in pyroptosis by stably silencing its expression in HeLa and HT29 human intestinal epithelial cells. As shown in new **Figure S3B-C**, silencing GBP2 has no effect on pyroptosis, supporting our conclusion that GBP1 is the key GBP involved in responses to infection with A/E pathogens.

2/ If GBP-1-dependent and indepndent recruitment of Casp4 to actin pedestal requires actin polymerization (either induced by bacteria or by the Ig-TIR constructs), one should expect that GBP1 and Casp4 would also target actin-comet tails from gram-positive bacteria Listeria monocytogenes or would also be able to target nacent phagosomes on macrophages as both are extremely enriched in polymerized actin structures that ressemble the pedestals (podosome-like structures or actin tails). These results would streghthen the author findings on GBP1 and Casp4 recruitment on these actin structures.

GBP1 does not target *Listeria* comets; we have therefore added the following to the Discussion (~line 393): "Similarly, GBP1 does not target the cytosolic actin comets of *Listeria monocytogenes*; in contrast, GBP1 targets membrane remnants following the escape of *Listeria* from its vacuole into the cytosol, pointing to exposed sugars from the vacuolar lumen as alternative signals that can attract GBP1 [1, 68]." Similarly, the mechanisms of actin polymerisation vary significantly among bacterial pathogens, which could also contribute to differential GBP1 targeting we have discussed further (line ~ 395): "The mechanisms of actin-based motility vary markedly among bacterial pathogens, which may underlie the lack of GBP1 targeting. For instance, A/E pathogens (Tir), *Shigella* (IcsA), *Burkholderia* (BimA), *Rickettsia* (RickA, Sca2), *Listeria* (ActA) differentially coopt actin nucleation promoting factors such as N-WASP, ARP2/3, ENA/VASP and others [69]."

Podosomes are actin-rich structures found predominantly in macrophages and not intestinal epithelia, and whether GBP1 targets these structures would indeed be of interest in a future study.

3/ The results that actin can recruit both GBP1 and Casp4 is of strong interest. Yet, at this step, there is a lack of discussion/ result about the putative function of GBP1 and Casp4 on actin structures that would be independent from the LPS-driven non canonical inflammasome response. I understand that authors might not have the experimental answer yet, but having some broader dicussion/speculation on this would be a strong added value for the study.

We thank the reviewer for inviting us to speculate – we have added the following text (~line 424): "The GBPs and caspase-4-like enzymes are conserved across vertebrate species, including domestic animals and pets that can be infected by A/E pathogens [73-76]. However, LPS-sensing is not universally conserved among caspase-4-related enzymes [74]. Similarly, whether GBP1-like proteins share LPS-sensing responses should also be assessed given the lineage-specific expansion and loss of the *GBP* gene cluster in vertebrates. The cytoskeleton is crucial for homeostasis and host defence [77] and a plethora of pathogens or their secreted toxins that hijack the actin cytoskeleton [78, 79]. The role of GBP1 in ETI against cytoskeleton manipulation and whether it responds to actin polymerisation in homeostasis are additional question to be addressed in the future."

Referee #2:

This manuscript by Shenoy and colleagues examines how extracellular A/E pathogens (EPEC, EHEC, Citrobacter rodentium) triggers pyroptosis in epithelial cells in humans and mice. Here, the authors demonstrate that bacterial-induced actin polymerisation triggers sequential recruitment of GBP1 and caspase-4 and downstream epithelial cell pyroptosis. While the observation that actin polymerisation is sufficient to induce GBP1 and caspase-4 recruitment in the absence of LPS is surprising, the overall conceptual advancement of the remaining manuscript is incremental (points 1, 2, 4) and contradictory to the authors' earlier work (point 3). I have also listed other suggestions for the authors' consideration (point 5 onwards).

1. The authors have already demonstrated in their 2019 Cell Reports paper (PMID: 31018119) that EPEC-induced actin polymerisation triggers caspase-4 activation in human macrophages, thus, demonstrating that the same pathway occurs in epithelial cells is not conceptually novel.

In our 2019 Cell Reports paper (PMID: 31018119) we reported how naïve human macrophage respond to **intracellular** EPEC, which we showed undergo **caspase-1-dependent pyroptosis**. In contrast, here we report novel LPS-independent sensing of **extracellular bacteria by IFNγ-treated intestinal epithelial cells**. Please also note that previous work did not investigate EHEC infection *in vitro* or *C .rodentium* infection *in vivo*. Therefore, PMID: 31018119 does not affect the novelty of this work (further described in pt 2).

2. The requirement for GBP1 to activate caspase-4 is well established and was demonstrated by the authors themselves and several other groups. Non-exhaustive list - PMID: 32783936, 31268602, 32581219, 32541830, 32510692, 37023136.

All of these are cited and indeed establish the 'canonical' role of GBP1-caspase-4 in **intracellular** Gram-ve bacteria sensing. This study mainly focuses on recruitment of GBP1 to the EPEC/EHEC adhesion sites. In contrast to all previous work on LPS/GBP1/caspase-4 we show that GBP1 and caspase-4 traffic to pedestals independently of LPS; moreover, recruited caspase-4 per se does not lead to activation – this is a strength and novelty of this study.

3. A previous study from these authors demonstrated that Tir clustering without downstream actin polymerisation is sufficient to trigger epithelial cell pyroptosis (PMID: 33378358), yet in this current manuscript the same group of authors report that actin polymerisation is required for GBP1 and caspase-4 recruitment and downstream pyroptosis. These results are contradictory to one another.

In PMID: 33378358 we used a highly modified EPEC strain that lacks all the T3SS effectors except Tir (EPEC-1), which while providing a powerful experimental tool, does not reflect physiological pathogen-host interactions. Here, we have used a range of prototypic and clinical EPEC and EHEC and *C. rodentium* which independently showed the role of Tir-mediated actin polymerisation in recruiting GBP1 and driving pyroptosis.

4. The most interesting (and novel) observation from this study is that GBP1 senses A/E-pathogen-induced actin polymerisation. However, this finding is mostly descriptive and the mechanism that drives GBP1 recruitment remains unclear.

While we and the other reviewers observe that the paper contained relevant mechanistic insight, we addressed this suggestion by adding significant amount of new data. We investigated trafficking and pyroptosis via GBP1 mutants that are defective in forming polymeric coats. Furthermore, we show that the prenylation-deficient C589A and GTP-binding defective K51A mutants do not traffic to pedestals, indicating that both properties of GBP1 are required for its trafficking. In contrast, LPS/membrane-binding and coatomer formation-defective ^{308}DLP^{310}→AAA (Kuhm, NSMB, 2025, 32:172) and M139D (Weismehl, EMBO J, 2025, 43:615) mutants found in recent cryo-EM studies can traffic to pedestals. This indicates that 'open' conformation parallel polymers formation and GTP hydrolysis may not contribute to GBP1 trafficking to pedestals. These data are shown in new Fig 4.

5. Majority of the work was done using HeLa cell, a transformed cervical epithelial cell line. Since A/E pathogens infect IECs, this work should be performed in IECs to reflect physiological relevance.

In addressing this comment, we now present new data using CL40 and HT29 cells as well as human colonic organoids. The results obtained with these cells mirrored those we recorded in HeLa cells, which validated our original approach. We thank the reviewer as these additional data have improved the manuscript. These data are shown in new Fig S2C-F, S3B-D.

6. I am assuming Hela (and IEC cell lines) do not express NLRP3 and thus IL-18 cleavage is caspase-4-dependent but not through secondary NLRP3-caspase-1 activation? This should be experimentally validated.

Correct. We confirmed that, in agreement with previous work, HeLa, HT29 and CL40 do not express NLRP3 (see image below), indicating that in these systems IL-18 maturation is driven by caspase-4. We have therefore added this sentence in the introduction (~line 64) "In macrophages, caspase-4 activation leads to downstream 'non-canonical' activation of the NLRP3 inflammasome leading to caspase-1-dependent maturation of IL-1β [19]".

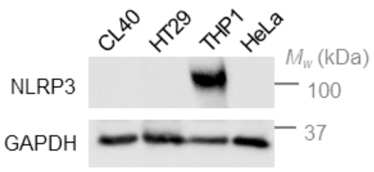

7. The authors demonstrated GBP1 and caspase-4-dependency in driving pyroptosis but has assumed in the manuscript that the downstream effector is GSDMD. Is this the case? This should be validated experimentally with GSDMD knock out and GSDMD cleavage.

Indeed, caspase-4 and GSDMD are involved in these processes; newly added data show inhibition of pyroptosis upon *CASP4* and/or *GSDMD* silencing in HeLa, CL40 and HT29 cells infected with EPEC (**new Fig S1A and S2D-E**).

8. The use of anti-LPS to detect Citrobacter rodentium in murine colon is far from ideal, since anti-LPS will pick up all Gram-negative bacteria from the colon. Using a more specific antibody will be more appropriate and convincing.

We apologise that the anti-CR antibody that we used was labelled as anti-LPS in legend to Figure 1, but correctly indicated as anti-CR in the Methods; this has now been corrected. Also see image below representative of sections from uninfected mice stained and imaged at the same time as data shown in Fig 1H from infected mice. Note that no staining is seen in CR and Gbp2 channels (actin staining is not detectable in uninfected cells at the low exposures required to see intense Phalloidin staining at CR pedestals)

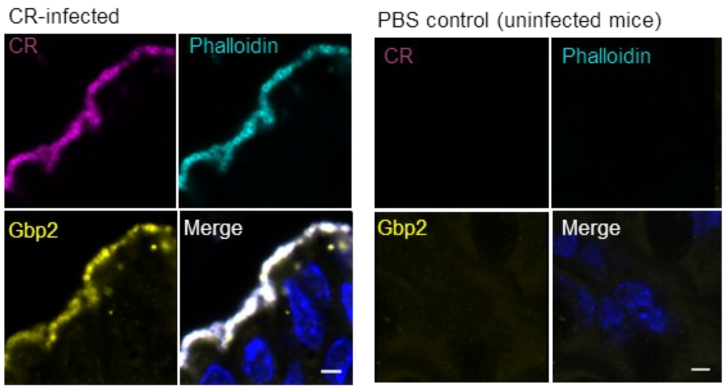

Referee #3:

The interferon-induced GTPase guanylate-binding protein 1 (GBP1) is well established as a key mediator of noncanonical inflammasome activation, specifically involving caspase-4 (CASP4) in humans. GBP1 has been shown to trigger CASP4 activation in response to infection with intracellular Gram-negative bacterial pathogens. Mechanistically, three principal models have been proposed, all converging on GBP1's capacity to interact with lipopolysaccharide (LPS):
i) GBP1 encapsulates Gram-negative bacteria in a detergent-like manner, promoting the release of LPS into the cytosol where it becomes available for CASP4 recognition
ii) GBP1 disrupts the O-antigen barrier, thereby exposing the lipid A moiety of the bacterial outer membrane to direct CASP4 binding
iii) GBP1 facilitates the aggregation of cytosolic LPS, forming high-affinity LPS platforms that enhance CASP4 recruitment.
However, these models have not adequately accounted for the phenomenon of CASP4 activation induced by extracellular Gram-negative bacteria. The present study addresses this gap and introduces a significant conceptual advance by revealing an LPS-independent mechanism of CASP4 recruitment mediated by actin pedestal formation.
Specifically, this study demonstrates that GBP1 is targeted to actin-pedestal structures formed by enteropathogenic (EPEC) and enterohaemorrhagic (EHEC) E. coli, and the EPEC-like murine pathogen Citrobacter rodentium, both in human cells and in vivo (mouse colonocytes). It is shown that GBP1 localizes to actin pedestals generated by Tir-Intimin signaling, a key virulence mechanism of EPEC/EHEC. Importantly, it is shown that this recruitment occurs independent of bacterial LPS. Using a chimeric receptor (FcR-Tir) that mimics Tir-driven actin polymerization without bacteria, the study proves that actin remodeling alone is sufficient to drive GBP1 recruitment, separating this process from LPS-dependent signaling. The findings extend GBP1's role from pattern recognition of cytosolic LPS to a novel effector-triggered immunity (ETI) function, where GBP1 acts as a sentinel for pathogen-induced actin cytoskeleton changes. The design of the studies is rigorous throughout. The paper is well written and flows logically. I don't see any notable weaknesses and am convinced that the paper will have a substantial impact on field.
While the study is already quite comprehensive, there appears to be a minor yet notable gap in the proposed model. The paper convincingly demonstrates that CASP4 is recruited to actin pedestals in an LPS-independent but GBP1-dependent manner.

A direct GBP1-CASP4 protein-protein interaction would provide the most straightforward mechanistic explanation for this observation. Although such experiments are not essential-given that the main conclusions are well supported by the presented data-it seems likely that the authors have considered, or perhaps even attempted, to explore this possibility. Some elaboration on this point, ideally supported by experimental evidence, would further strengthen the manuscript. In the same vein, it would be valuable for the authors to discuss their findings in the context of previous studies demonstrating GBP1-CASP4 co-recruitment to cytosolic Gram-negative bacteria. The prevailing view posits that GBP1 coating exposes the lipid A portion of LPS, thereby enabling CASP4 binding. However, in light of the current findings, an alternative model - where GBP1 directly recruits CASP4 to bacterial surfaces independently of LPS - merits consideration.

Thank you for these suggestions. Indeed, GBP1 and caspase-4 have been previously shown to form a complex in IFNγ-treated cells by us (Fisch, Cell Rep, 2019, EMBO J, EMBO J. 2019, 38:e100926), as well as the Randow lab (Wandel Nat Immunol, 2020 Nat Immunol. 21:880). It is for this reason that we decided instead to focus on GBP1 trafficking and whether LPS was responsible for this activity. In light of our findings, we have suggested a 3-step process in the Discussion (line ~326 onwards): "Here we found that actin polymerisation by Tir recruits GBP1 to pedestals, which is followed by caspase-4 for LPS-sensing at bacterial attachment sites. We and others have previously showed that GBP1 and caspase-4 form a complex in response to bacterial infection or LPS transfection [9-12], therefore they may also interact during EPEC/EHEC infection leading to their localization to bacterial pedestals. Further, murine caspase-4/11 is proposed to localise to the leading edge in leukocytes; therefore, the diffuse caspase-4 staining at EPEC/EHEC pedestals in *GBP1*$^{-/-}$ cells may point to its ability to traffic to the plasma membrane in homeostasis [57]. We propose that the pyroptosis induced by extracellular A/E pathogens proceeds via three steps: (i) trafficking of GBP1 to actin-rich pedestals to 'prime' the response, (ii) recruitment of caspase-4 for LPS-sensing, and (iii) stimulation of caspase-4 activity leading to pyroptosis and cytokine maturation (***Figure 5F***)."

Lastly, it would be interesting to discuss whether GBP1 recognition of pathogen-containing vacuoles (PVs) might similarly lead to CASP4 recruitment, or whether GBP1 interactions at actin pedestals represent a fundamentally distinct mode of CASP4 engagement.

This is an interesting point – we have added the following to the Discussion (~line 370). "Whether GBP1-dependent caspase-4 recruitment to Tir-driven structures is fundamentally distinct from its recruitment to the surface of *Salmonella/Shigella* awaits further investigation. GBP1 can detect LPS-free intact pathogen-containing vacuoles (e.g., *Toxoplasma*), which could serve as a model to dissect whether GBP1 can recruit caspase-4 to other intracellular pathogen niches. However, stimulation of caspase-4 activity would still require LPS; therefore, any non-canonical roles for caspase-4 in such scenarios also remain to be identified [65]."

Dear Avinash,

Thank you again for the submission of your revised manuscript (EMBOJ-2025-122562R-Q) to The EMBO Journal for our consideration, and for your patience during re-review. As I have already informed you, your revision has been seen by the three original referees, who had previously assessed the first version of your manuscript, and we have received their comments, which I have shared with you (they are appended again below). I am pleased to say that all three referees are satisfied with the revision and recognize that the initially raised criticisms and concerns have been sufficiently addresssed. I am therefore happy to inform you that your manuscript has been accepted in principle for publication in The EMBO Journal.

Before we can proceed with the next steps towards publication of your article, there are a few changes and corrections we need you to make in a final resubmission:

- The main manuscript file should be uploaded in .docx (instead of .pdf) file format; the Figures should be removed from that file and uploaded instead as individual, production-quality Figure files; their legends should remain in the manuscript file, placed below the References list.

- Please enter the details of all co-authors in our online manuscript handling system; co-authors Ayush Punwatkar, Miyu Stephenson, Sandra Koigi, Diana Papp, John P Thomas, Dimple Dixit, and Tamas Korcsmaros are currently missing from the online system.

- The corresponding author must be marked (e.g., with an asterisk) in the authors' list on the title page of the manuscript.

- Please note that our notifications could not be delivered to co-author Daniel J. Bennison (at d.bennison@imperial.ac.uk); please make sure that his profile in our system is up-to-date with a valid e-mail address, or send us this information so that we can update it for you.

- Please note that all funding information provided in the Acknowledgements section of the manuscript should also be provided in our online manuscript handling system; no such information has been entered in our online system yet.

- The reference format must be updated to the style used by EMBO Press (i.e. alphabetical list, with the names of the first 10 co-authors listed for each citation, followed by "et al."). For more information, please refer to our Author Guidelines: https://link.springer.com/journal/44318/submission-guidelines#cms-Reference-guidelines.

- Before submitting your revision, primary datasets (and computer code, where appropriate) produced in this study need to be deposited in appropriate public databases (see https://link.springer.com/partners/embo-press/editorial-policies#Data%20availability%20statement). In particular, all datasets such as DNA or RNA sequencing data, or mass spectrometry data generated in the study must be deposited in appropriate repositories. The accession numbers, database, and the specific URLs (links) should be listed in a formal "Data availability" section (placed after Methods), following the example below:

"The RNA-seq datasets produced in this study are available in the following database:
Gene Expression Omnibus GSE46843 (https://www.ncbi.nlm.nih.gov/geo/query/acc.cgi?acc=GSE46843)"

*** All links should resolve to a page where the data can be accessed. ***
*** Please remember to provide in the Data availability section of your revised manuscript reviewer passwords if the datasets are not yet public. ***
*** The Data Availability Section is restricted to new primary data that are part of this study. In case you have no data that require deposition in a public database, please state so instead of referring to the database: "Our study includes no data deposited in public repositories." under the heading "Data availability". ***

- We kindly request that authors consider both actual and perceived competing interests. Please review our policy (https://link.springer.com/partners/embo-press/editorial-policies#Competing%20interest%20disclosures) and include a competing interests statement with heading "Disclosure and competing interests statement", placed after the Acknowledgements section of the manuscript.

- We use CRediT to specify the contributions of each author in the journal submission system. CRediT replaces the author contribution section, which should be removed from the manuscript. Please use the free text box to provide more detailed descriptions. See also guide to authors for more information: https://link.springer.com/partners/embo-press/editorial-policies#Authorship.

- We noticed that callouts for Fig. 1H are missing.

- Please include in your resubmission a completed author checklist, which you can download from our author guidelines (https://link.springer.com/journal/44318/submission-guidelines). Please note that the checklist will also be part of the Peer Review File that will be published alongside your article.

- The Appendix (currently labeled "Supplementary Information" in the manuscript) should be removed from the main manuscript file and instead uploaded as an individual PDF file; please include heading "Appendix for:" on its title page, followed by the manuscript's title (author and affiliation list are not necessary) and a "Table of Contents" including page numbers for all listed items; the nomenclature for all Appendix items should be "Appendix Figure S#" and "Appendix Table S#" throughout the manuscript and the Appendix file.

- The materials and methods need to be described in the manuscript using our structured methods format, which is now required for all research articles. According to this format, the Methods section includes a single "Reagents and Tools Table" -listing key reagents, oligos, experimental models, software and relevant equipment including their sources and relevant identifiers- followed by a "Methods and Protocols" section describing the methods. Please download and fill in our Reagents and Tools Table template (.docx), which you can find in our author guide: https://link.springer.com/journal/44318/submission-guidelines#cms-Manuscript-organisation-and-formatting. When submitting your revised manuscript, please do not include the Reagents and Tools Table in the Methods section of the manuscript but instead upload it as a separate file choosing the file type "Reagent Table" (not in PDF format).

- Heading "Materials & Methods" should be updated to "Methods".

- "Ethics Statement" should be incorporated in the "Methods" section.

- At EMBO Press we ask authors to provide source data for the main manuscript figures. You will receive a separate email with instructions for providing source data with your revised manuscript, including how to upload and organize the files.

- Please note that EMBO press papers are accompanied online by:
A) a short (2 sentences) summary of the findings and their significance,
B) 2-5 short bullet points highlighting the key results, and
C) a synopsis image in .jpg or .png format that is exactly 550 pixels wide and 300 pixels high. Please note that all text needs to be legible at the final size.
Please upload this information along with your revised manuscript (the text for A and B should be provided in a separate Word file).

- Our data editors have checked your Figures and their legends, and raised the following queries. Please address all points below completely in your revised manuscript (all changes should be highlighted or "tracked" in the Word file):
1. Please provide the exact p-values in the legends of Figures 1A-C; 2B,D; 3A,B; 4A,C; 5E; S1-A,B,E; S2-D,E; S3-C,D; S4-B.
2. Please note that information related to "n" is missing in the legends of Figures S1-B.
3. Please note that the error bars should be defined in the legend of Figure S1-B.

- The manuscript sections need to be named and ordered as follows: Title page - Abstract - Introduction - Results - Discussion - Methods - Data Availability - Acknowledgements - Disclosure and Competing Interests Statement - References - Figure Legends - main Tables (if there are any) - Expanded View Figure Legends (if there are any).

- Please also note that as part of the EMBO Press transparent editorial process, The EMBO Journal publishes online a Peer Review File along with each accepted manuscript. This File will be published in conjunction with your paper and will include the referee reports, your point-by-point responses and all pertinent correspondence relating to the manuscript. Your Author's Checklist will also be published at the end of the Peer Review File. Please let us know in case you want to remove any data or figures from your point-by-point responses before they are published as part of the Peer Review File. Retaining unpublished data in the Peer Review File means that these count as published and that the Peer Review File would need to be referenced in future publications. Please let the editorial office know in case you want to remove any data from this file (contact@embojournal.org).

We look forward to seeing a final version of your manuscript as soon as possible. Please let us know if you have any questions and use this link to submit your revision: https://emboj.msubmit.net/cgi-bin/main.plex.

Best regards,

Ioannis

Ioannis Papaioannou, PhD

Editor, The EMBO Journal
i.papaioannou@embojournal.org

Referee #1:

In their rebuttal letter the authors tried experimentally and by discussing to adress the two major critics that the reviewers raised. The first one was about the role of GBP1 at driving Casp4 recruitment and its the putative involvement at activating Casp4 on those pedestals. This has been answered in the rebuttal letter. Although some questions remain regarding the clear ability of GBP1 at driving Casp4 activation specifically in those pedestals, the data provided support the observations of the authors. Regarding the GBP1-independent role in recruiting Casp4, the proposed explanation remains quite blurry, but I understand that the authors might not have the experimental rational about it.

The second critic was about the lack of clear mechanistic involved in such process. At this step, the authors showed that some mutants of GBP1 were impaired in their recruitment to the pedestals. Those mutants however, overlap with their ability to activate Caspase-4 in response to LPS, hence impeding adressing a clear mechanistic study.
Yet, at this step, this is something that will probably be adressed in furture studies.

At this step, the study is of importance and bring scientific novelty despite its very descriptive form.

Referee #2:

The authors have addressed my comments appropriately.
Congratulations on a nice study.

Referee #3:

My concerns were sufficiently addressed.

All editorial and formatting issues were resolved by the authors.

1st Revision - Editorial Decision **15th May 2026**

Dear Avinash,

Congratulations on an excellent manuscript! I am very pleased to inform you that it has been accepted for publication in The EMBO Journal. Thank you for comprehensively addressing the initially raised referees' concerns and our editorial requests for corrections and changes.

Your manuscript will be processed for publication by EMBO Press. It will be copy edited and you will receive page proofs prior to publication. Please note that you will be contacted by Springer Nature Author Services to complete licensing and payment information.

You may qualify for financial assistance for your publication charges - either via a Springer Nature fully open access agreement or an EMBO initiative. Check your eligibility: https://link.springer.com/journal/44318/how-to-publish-with-us

Should you be planning a Press Release on your article, please get in contact with embo_production@springernature.com as early as possible in order to coordinate publication and release dates.

If you have any questions, please do not hesitate to contact the Editorial Office. Thank you for your contribution to The EMBO Journal. Working with you has been a pleasure.

Best regards,

Ioannis

Ioannis Papaioannou, PhD
Editor, The EMBO Journal
i.papaioannou@embojournal.org

*****
Please note that it is The EMBO Journal policy for the transcript of the editorial process (containing referee reports and your response letters) to be published as an online supplement to each paper. If you should prefer removal of any referee-only figures included in the point-by-point response(s), e.g. because they may still be used for future publication or because they have been reproduced from published work by others, please do let us know immediately via response email.
More information is available here: https://link.springer.com/partners/embo-press/editorial-policies#Peer%20review

** Click here to be directed to your login page: https://emboj.msubmit.net

EMBO Press Author Checklist

Corresponding Author Name: Avinash R Shenoy	
Journal Submitted to: The EMBO Journal	
Manuscript Number: EMBOJ-2025-122562R-Q	

USEFUL LINKS FOR COMPLETING THIS FORM
The EMBO Journal - Author Guidelines
EMBO Reports - Author Guidelines
Molecular Systems Biology - Author Guidelines
EMBO Molecular Medicine - Author Guidelines

Reporting Checklist for Life Science Articles (updated January
This checklist is adapted from Materials Design Analysis Reporting (MDAR) Checklist for Authors. MDAR establishes a minimum set of requirements in transparent reporting in the life sciences (see Statement of Task: 10.31222/osf.io/9sm4x). Please follow the journal's guidelines in preparing your
Please note that a copy of this checklist will be published alongside your article.

Abridged guidelines for figures
1. Data
The data shown in figures should satisfy the following conditions:
- the data were obtained and processed according to the field's best practice and are presented to reflect the results of the experiments in an accurate and unbiased manner.
- ideally, figure panels should include only measurements that are directly comparable to each other and obtained with the same assay.
- plots include clearly labeled error bars for independent experiments and sample sizes. Unless justified, error bars should not be shown for technical
- if n<5, the individual data points from each experiment should be plotted. Any statistical test employed should be justified.
- Source Data should be included to report the data underlying figures according to the guidelines set out in the authorship guidelines on Data

2. Captions
Each figure caption should contain the following information, for each panel where they are relevant:
- a specification of the experimental system investigated (eg cell line, species name).
- the assay(s) and method(s) used to carry out the reported observations and measurements.
- an explicit mention of the biological and chemical entity(ies) that are being measured.
- an explicit mention of the biological and chemical entity(ies) that are altered/varied/perturbed in a controlled manner.
- the exact sample size (n) for each experimental group/condition, given as a number, not a range;
- a description of the sample collection allowing the reader to understand whether the samples represent technical or biological replicates (including how many animals, litters, cultures, etc.).
- a statement of how many times the experiment shown was independently replicated in the laboratory.
- definitions of statistical methods and measures:
  - common tests, such as t-test (please specify whether paired vs. unpaired), simple χ2 tests, Wilcoxon and Mann-Whitney tests, can be unambiguously identified by name only, but more complex techniques should be described in the methods section;
  - are tests one-sided or two-sided?
  - are there adjustments for multiple comparisons?
  - exact statistical test results, e.g., P values = x but not P values < x;
  - definition of 'center values' as median or average;
  - definition of error bars as s.d. or s.e.m.

Please complete ALL of the questions below.
Select "Not Applicable" only when the requested information is not relevant for your study.

Materials

Newly Created Materials	Information included in the manuscript?	In which section is the information available? (Reagents and Tools Table, Materials and Methods, Figures, Data Availability Section)
New materials and reagents need to be available; do any restrictions apply?	Yes	Reagents and Tools Table

Antibodies	Information included in the manuscript?	In which section is the information available? (Reagents and Tools Table, Materials and Methods, Figures, Data Availability Section)
For **antibodies** provide the following information:<br>- Commercial antibodies: RRID (if possible) or supplier name, catalogue number and or/clone number<br>- Non-commercial: RRID or citation	Yes	Reagents and Tools Table

DNA and RNA sequences	Information included in the manuscript?	In which section is the information available? (Reagents and Tools Table, Materials and Methods, Figures, Data Availability Section)
**Short novel DNA or RNA including primers, probes**: provide the sequences.	Yes	Reagents and Tools Table

Cell materials	Information included in the manuscript?	In which section is the information available? (Reagents and Tools Table, Materials and Methods, Figures, Data Availability Section)
**Cell lines:** Provide species information, strain. Provide accession number in repository **OR** supplier name, catalog number, clone number, and/**OR** RRID.	Yes	Reagents and Tools Table
**Primary cultures:** Provide species, strain, sex of origin, genetic modification status.	Not Applicable	
Report if the cell lines were recently **authenticated** (e.g., by STR profiling) and tested for mycoplasma contamination.	Yes	Methods

Experimental animals	Information included in the manuscript?	In which section is the information available? (Reagents and Tools Table, Materials and Methods, Figures, Data Availability Section)
**Laboratory animals or Model organisms:** Provide species, strain, sex, age, genetic modification status. Provide accession number in repository **OR** supplier name, catalog number, clone number, **OR** RRID.	Not Applicable	
**Animal observed in or captured from the field:** Provide species, sex, and age where possible.	Not Applicable	
Please detail **housing and husbandry conditions**.	Not Applicable	

Plants and microbes	Information included in the manuscript?	In which section is the information available? (Reagents and Tools Table, Materials and Methods, Figures, Data Availability Section)
**Plants:** provide species and strain, ecotype and cultivar where relevant, unique accession number if available, and source (including location for collected wild specimens).	Not Applicable	
**Microbes:** provide species and strain, unique accession number if available, and source.	Yes	Reagents and Tools Table

Human research participants	Information included in the manuscript?	In which section is the information available? (Reagents and Tools Table, Materials and Methods, Figures, Data Availability Section)
If collected and within the bounds of privacy constraints report on age, sex and gender or ethnicity for all study participants.	Not Applicable	

Core facilities	Information included in the manuscript?	In which section is the information available? (Reagents and Tools Table, Materials and Methods, Figures, Data Availability Section)
If your work benefited from core facilities, was their service mentioned in the acknowledgments section?	Yes	Acknowledgements

Design

Study protocol	Information included in the manuscript?	In which section is the information available? (Reagents and Tools Table, Materials and Methods, Figures, Data Availability Section)
If study protocol has been **pre-registered, provide DOI in the manuscript**. For clinical trials, provide the trial registration number **OR** cite DOI.	Not Applicable	
Report the **clinical trial registration number** (at ClinicalTrials.gov or equivalent), where applicable.	Not Applicable	

Laboratory protocol	Information included in the manuscript?	In which section is the information available? (Reagents and Tools Table, Materials and Methods, Figures, Data Availability Section)
Provide DOI OR other citation details if **external detailed step-by-step protocols** are available.	Not Applicable	

Experimental study design and statistics	Information included in the manuscript?	In which section is the information available? (Reagents and Tools Table, Materials and Methods, Figures, Data Availability Section)
Include a statement about **sample size** estimate even if no statistical methods were used.	Yes	Methods
Were any steps taken to minimize the effects of subjective bias when allocating animals/samples to treatment (e.g. **randomization procedure**)? If yes, have they been described?	Yes	Methods
Include a statement about **blinding** even if no blinding was done.	Yes	Methods
Describe **inclusion/exclusion criteria** if samples or animals were excluded from the analysis. Were the criteria pre-established?<br><br>If sample or data points were omitted from analysis, report if this was due to attrition or intentional exclusion and provide justification.	Yes	Methods
For every figure, are **statistical tests** justified as appropriate? Do the data meet the assumptions of the tests (e.g., normal distribution)? Describe any methods used to assess it. Is there an estimate of variation within each group of data? Is the variance similar between the groups that are being statistically compared?	Yes	Methods

Sample definition and in-laboratory replication	Information included in the manuscript?	In which section is the information available? (Reagents and Tools Table, Materials and Methods, Figures, Data Availability Section)
In the figure legends: state number of times the experiment was **replicated** in laboratory.	Yes	Figure Legends
In the figure legends: define whether data describe **technical or biological replicates**.	Yes	Figure Legends and Methods

Ethics

Ethics	Information included in the manuscript?	In which section is the information available? (Reagents and Tools Table, Materials and Methods, Figures, Data Availability Section)
Studies involving **human participants**: State details of **authority granting ethics approval** (IRB or equivalent committee(s), provide reference number for approval.	Yes	Methods
Studies involving **human participants**: Include a statement confirming that **informed consent** was obtained from all subjects and that the experiments conformed to the principles set out in the WMA Declaration of Helsinki and the Department of Health and Human Services Belmont Report.	Yes	Methods
Studies involving **human participants:** For publication of **patient photos**, include a statement confirming that consent to publish was obtained.	Not Applicable	
Studies involving experimental **animals**: State details of **authority granting ethics approval** (IRB or equivalent committee(s), provide reference number for approval. Include a statement of compliance with ethical regulations.	Yes	Methods
Studies involving **specimen and field samples:** State if relevant **permits** obtained, provide details of authority approving study; if none were required, explain why.	Not Applicable	

Dual Use Research of Concern (DURC)	Information included in the manuscript?	In which section is the information available? (Reagents and Tools Table, Materials and Methods, Figures, Data Availability Section)
Could your study fall under dual use research restrictions? Please check biosecurity documents and list of **select agents and toxins** (CDC): https://www.selectagents.gov/sat/list.htm	Not Applicable	
If you used a select agent, is the security level of the lab appropriate and reported in the manuscript?	Not Applicable	
If a study is subject to dual use research of concern regulations, is the name of the **authority granting approval and reference number** for the regulatory approval provided in the manuscript?	Not Applicable	

Reporting

The MDAR framework recommends adoption of discipline-specific guidelines, established and endorsed through community initiatives. Journals have their own policy about requiring specific guidelines and recommendations to complement MDAR.

Adherence to community standards	Information included in the manuscript?	In which section is the information available? (Reagents and Tools Table, Materials and Methods, Figures, Data Availability Section)
State if relevant guidelines or checklists (e.g., **ICMJE, MIBBI, ARRIVE, PRISMA**) have been followed or provided.	Not Applicable	
For **tumor marker prognostic studies,** we recommend that you follow the **REMARK** reporting guidelines (see link list at top right). See author guidelines, under 'Reporting Guidelines'. Please confirm you have followed these guidelines.	Not Applicable	
For **phase II and III randomized controlled trials**, please refer to the **CONSORT** flow diagram (see link list at top right) and submit the CONSORT checklist (see link list at top right) with your submission. See author guidelines, under 'Reporting Guidelines'. Please confirm you have submitted this list.	Not Applicable	

Data Availability

Data availability	Information included in the manuscript?	In which section is the information available? (Reagents and Tools Table, Materials and Methods, Figures, Data Availability Section)
Have **primary datasets** been deposited according to the journal's guidelines (see 'Data Deposition' section) and the respective accession numbers provided in the Data Availability Section?	Yes	Data Availability Section
Were **human clinical and genomic datasets** deposited in a public access-controlled repository in accordance to ethical obligations to the patients and to the applicable consent agreement?	Not Applicable	
Are **computational models** that are central and integral to a study available without restrictions in a machine-readable form? Were the relevant accession numbers or links provided?	Not Applicable	
If publicly available data were reused, provide the respective **data citations in the reference list**.	Not Applicable	
